# Induction of Apoptosis and Subsequent Phagocytosis of Virus-Infected Cells As an Antiviral Mechanism

**DOI:** 10.3389/fimmu.2017.01220

**Published:** 2017-09-28

**Authors:** Firzan Nainu, Akiko Shiratsuchi, Yoshinobu Nakanishi

**Affiliations:** ^1^Laboratory of Pharmacology and Toxicology, Faculty of Pharmacy, Hasanuddin University, Makassar, Indonesia; ^2^Laboratory of Host Defense and Responses, Graduate School of Medical Sciences, Kanazawa University, Kanazawa, Japan

**Keywords:** viral infection, apoptosis, phagocytosis, innate immunity, antiviral mechanism

## Abstract

Viruses are infectious entities that hijack host replication machineries to produce their progeny, resulting, in most cases, in disease and, sometimes, in death in infected host organisms. Hosts are equipped with an array of defense mechanisms that span from innate to adaptive as well as from humoral to cellular immune responses. We previously demonstrated that mouse cells underwent apoptosis in response to influenza virus infection. These apoptotic, virus-infected cells were then targeted for engulfment by macrophages and neutrophils. We more recently reported similar findings in the fruit fly *Drosophila melanogaster*, which lacks adaptive immunity, after an infection with *Drosophila* C virus. In these experiments, the inhibition of phagocytosis led to severe influenza pathologies in mice and early death in *Drosophila*. Therefore, the induction of apoptosis and subsequent phagocytosis of virus-infected cells appear to be an antiviral innate immune mechanism that is conserved among multicellular organisms. We herein discuss the underlying mechanisms and significance of the apoptosis-dependent phagocytosis of virus-infected cells. Investigations on the molecular and cellular features responsible for this underrepresented virus–host interaction may provide a promising avenue for the discovery of novel substances that are targeted in medical treatments against virus-induced intractable diseases.

## Introduction

Viruses are one of the most abundant entities present in the environment. All species, including microbial pathogens, such as bacteria and fungi, are subject to infections by viruses (1, 2). Greater susceptibility to viral infection has been reported in higher metazoans, such as humans, which live in a community system (3). In this system, close interactions exist between species, and, thus, infection easily spreads among members of the community (3), particularly under the condition of compromised immunity (4). Irrespective of the types of genomes and other structural and functional characteristics, viruses behave in a similar manner after invading host organisms. Most viruses, if not all, are obligate intracellular parasites and, thus, require immediate access to the cytosolic and/or nuclear compartments of host cells (2, 5). In the cytoplasm, viruses hijack the ribosomes of host cells to generate proteins encoded by their own genomes for the production of new infective virions (5–8). In the nucleus, viruses may utilize, when necessary, host enzymes to replicate their genomes and synthesize mRNA. On the other hand, host cells are equipped with an array of intracellular and extracellular immune responses to limit this viral proliferation process (9, 10). The final result of this race between the host and virus decides the outcome of infection, from which infected host organisms become ill or remain healthy. Although drugs have been developed to combat diseases caused by viral infections, their efficacy, unlike those against bacteria and fungi, is limited to certain types of viruses: targets for effective drugs are nucleoside kinases of herpes virus, protease and reverse transcriptase of human immunodeficiency virus, neuraminidase of influenza virus, and non-structural proteins of hepatitis C virus (11–13). Some infectious diseases, such as those caused by Ebola virus and highly pathogenic influenza virus, have been challenging to treat and often result in a large number of deaths (14–17). Therefore, medical treatments that are effective against different types of viruses are urgently required.

We herein highlight an underrepresented virus–host interaction, the apoptosis-dependent phagocytosis of virus-infected cells, which enables the elimination of viruses as an innate immune response. This mechanism may be effective against most types of viruses and appears to be conserved among multicellular organisms. Therefore, it may provide a better rationale for the development of novel medical treatments against virus-induced diseases (18, 19).

## Phagocytic Elimination of Cells Undergoing Apoptotic Death

**Figure 1:**
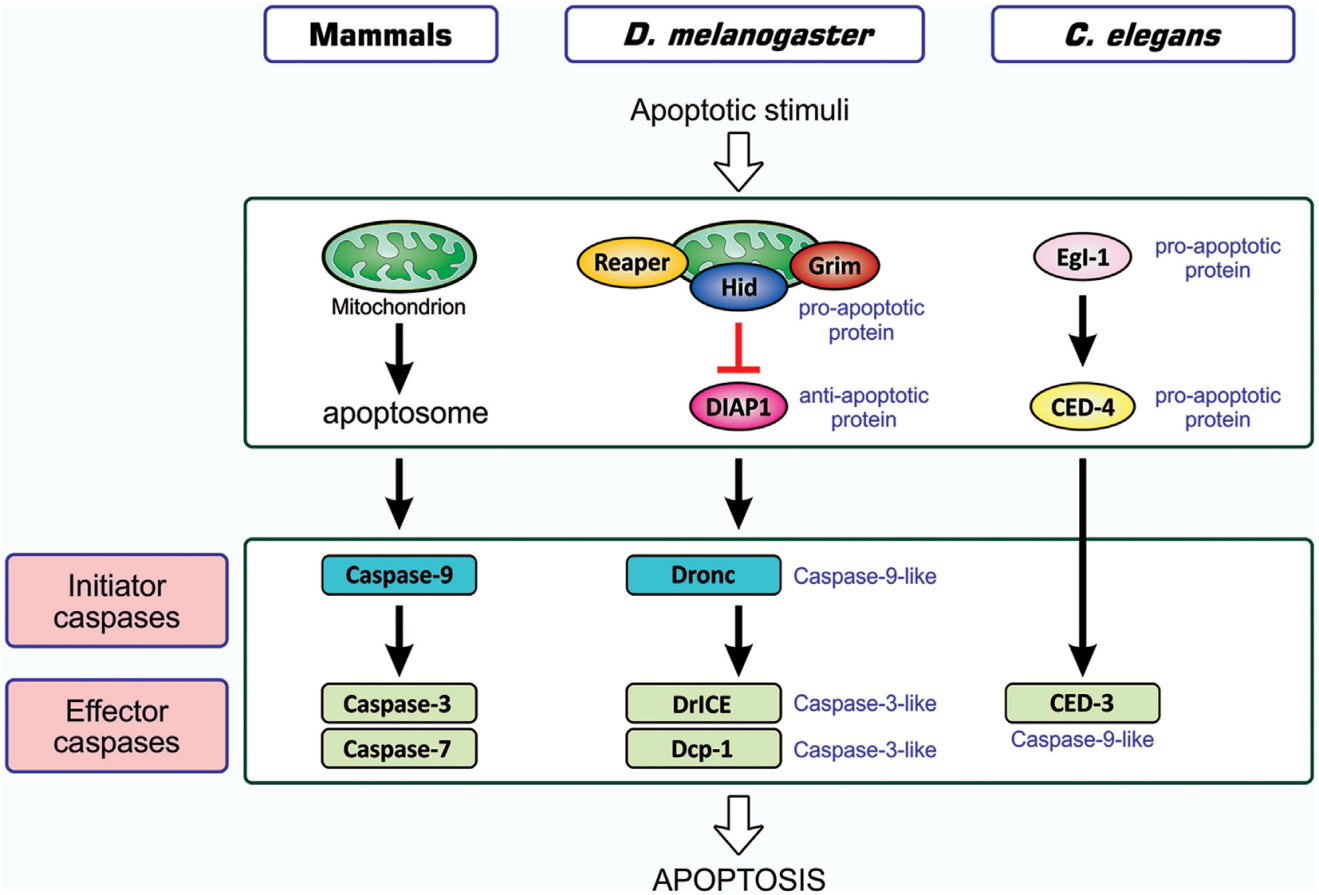
Similarity in apoptosis-inducing pathways in three model animals. Pathways for the induction of apoptosis in mammals, *Drosophila melanogaster*, and *Caenorhabditis elegans* are shown. Not all signal mediators are indicated. In the activation of initiator caspases, the mitochondrion is involved in mammals, probably involved in *Drosophila*, and not involved in *C. elegans*. Initiator caspases are caspase-9 in mammals, caspase-9-like Dronc in *Drosophila*, and absent in *C. elegans*, while effector caspases are caspase-3 and caspase-7 in mammals, caspase-3-like DrICE and Dcp-1 in *Drosophila*, and caspase-9-like CED-3 in *C. elegans*. Effector caspases, once activated by initiator caspases, degrade a number of cellular proteins, leading to structural and functional features that are typical of apoptosis. In mammals, two different modes of apoptosis-inducing pathways exist, and the so-called intrinsic pathway that involves the action of mitochondria is shown. The other one, the extrinsic pathway, which is initiated by extracellular death factors and their receptors independent of mitochondrial actions, is not shown (see Figure 4). CED, cell death abnormal; Dcp-1, death caspase-1; DrICE, *Drosophila* interleukin-1 β-converting enzyme; Dronc, *Drosophila* Nedd2-like caspase; Egl-1, egg-laying defective-1.

As one of the host responses evoked upon viral infection, host cells are induced to undergo apoptotic death (20, 21). Apoptosis is an orchestrated process of self-demolition, which is observed across metazoan species and considered to be a major form of programmed cell death (22–24). The pathways for the induction of apoptosis have been documented for three model animals—the nematode *Caenorhabditis elegans*, the fruit fly *Drosophila melanogaster*, and the mouse *Mus musculus*—and are shown to be fundamentally equivalent (22, 25, 26), as illustrated in Figure 1. All cellular changes observed during the apoptotic process are generally attributed to the actions of cysteine-proteases, caspases, and the onset of apoptosis involves the activation of initiator caspases that, in turn, partially cleave and activate another group of caspases, the effector caspase (24, 27, 28). Activated effector caspases then cleave a number of cellular proteins, resulting in the structural and biochemical features of apoptosis, such as the shrinkage of cells, fragmentation of DNA, and condensation of chromatin (22).

Apoptotic cells completely disappear: they are engulfed and digested by immune cells, a process-dubbed phagocytosis (29–31). Phagocytosis was described more than 100 years ago through the seminal studies of the late professor Elie Metchnikoff (32, 33). Researchers initially investigated the mechanisms underlying the phagocytosis of microbial pathogens that invade the human body and later identified apoptotic cells as another target. The phagocytosis of microbial pathogens is evident in innate and adaptive immune responses: phagocytes bind to surface structures specific to the target in the former response, while immunoglobulin, which binds antigens on the surface of pathogens and is often called an opsonin, functions as a ligand for an engulfment receptor, i.e., the Fc receptor, of phagocytes in the adaptive response. In contrast, antibodies are not involved in the phagocytosis of apoptotic cells, and the surface structures of the target that undergo modifications during the apoptotic process are recognized by the engulfment receptors of phagocytes. Under certain conditions, soluble proteins called bridging molecules connect apoptotic cells and phagocytes, similar to opsonins; however, these bridging molecules do not include immunoglobulin. Therefore, apoptotic cell clearance is categorized into an innate immune response to eliminate cells unwanted by the body. The phagocytic elimination of cells undergoing apoptosis is so rapidly accomplished that apoptotic cells are rarely detected in tissues and organs. Cells undergoing apoptosis maintain the integrity of plasma membrane permeability until engulfment by phagocytes, and thus the noxious components of cells do not leak out and damage surrounding tissues. Therefore, apoptosis is considered to be a physiological, silent mode of cell death (34, 35).

**Figure 2:**
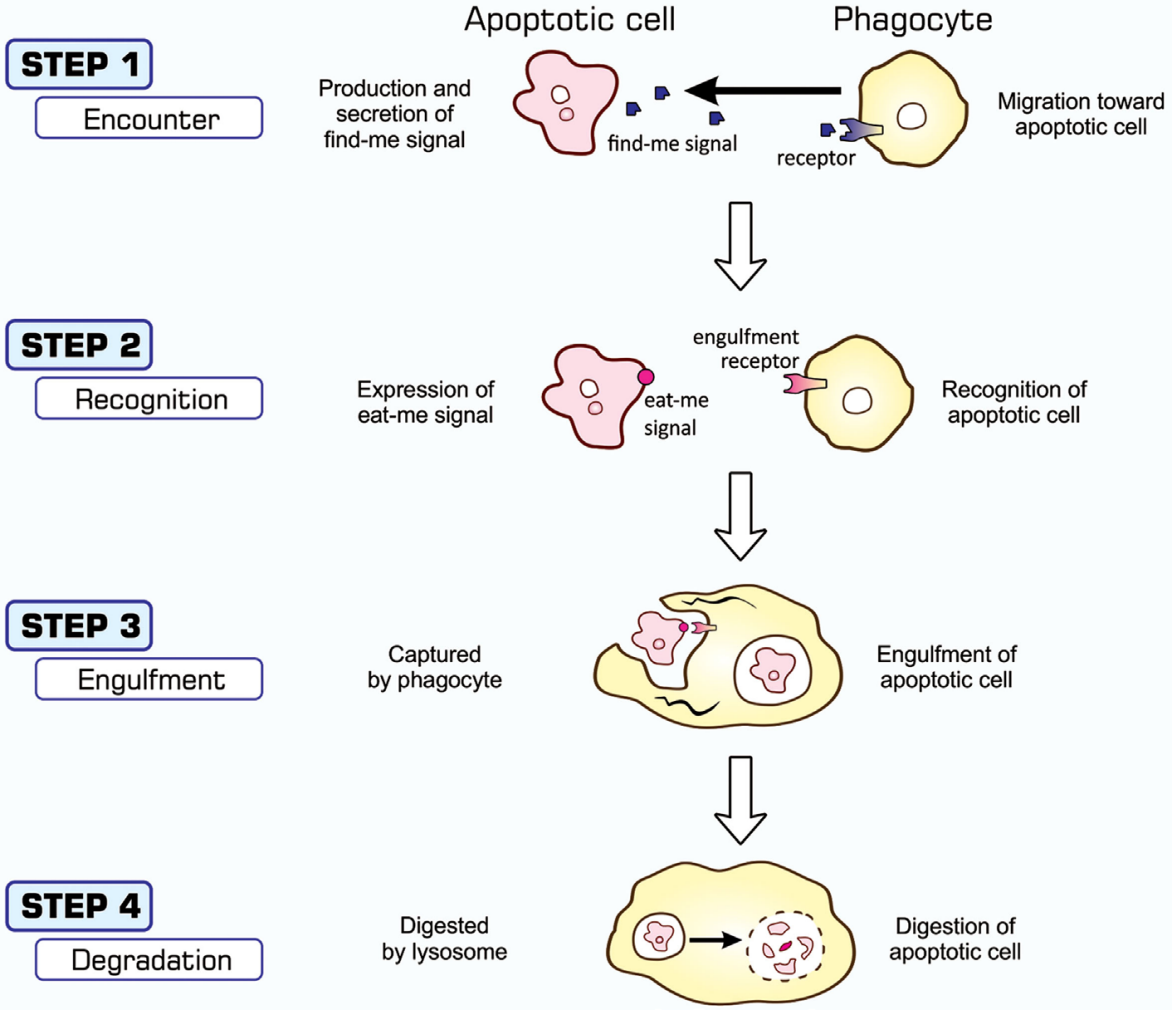
Processes of apoptosis-dependent phagocytosis. Cells undergoing apoptosis secrete substances that attract phagocytes (STEP 1), often called find-me signals, and simultaneously express eat-me signals on their surface (STEP 2). Phagocytes that come into close proximity to apoptotic cells recognize and bind eat-me signals using engulfment receptors (STEP 2), and activate signaling pathways for the induction of phagocytosis. The culmination of this signal transduction is the generation of pseudopodia that help phagocytes surround and incorporate apoptotic cells (STEP 3). Materials engulfed exist as phagosomes, which subsequently fuse with the lysosomes for degradation (STEP 4).

**Figure 3:**
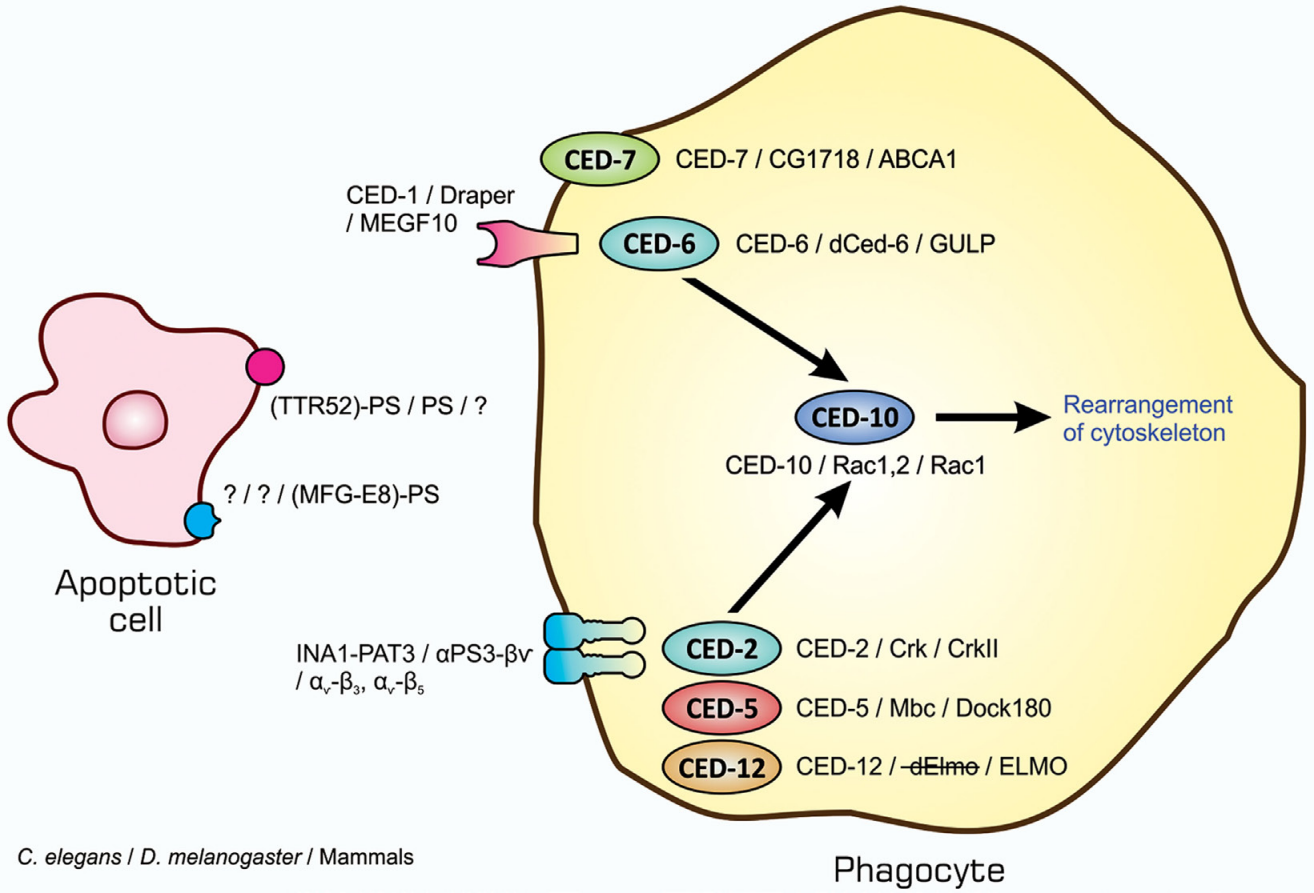
Similarity in signaling pathways for the induction of apoptotic cell clearance in three model animals. Molecules constituting two partly overlapping pathways for the induction of phagocytosis in the nematodes, insects, and mammals are shown. The names of eat-me signals, bridging molecules, engulfment receptors, and intracellular signal mediators of *Caenorhabditis elegans, Drosophila melanogaster*, and mammals are shown from left to right with slashes in between. All molecules in each individual category are counterparts to each other in three animal species. Refer to the text for explanation.

The entire process of the phagocytosis of apoptotic cells is shown in Figure 2. Apoptotic cells that are close to engulfment release substances, which are often referred to as find-me signals, to recruit phagocytes. A number of molecules have been reported to act as such signals, including proteins, lipids, and nucleotides, and their receptors as well as downstream signal transduction pathways have been mostly identified (36–38). Phagocytosis is initiated when apoptotic cells are in close proximity to phagocytes, which allows engulfment receptors on the surface of phagocytes to recognize and bind to ligands on the surface of target apoptotic cells (34, 35). The ligands for engulfment receptors are called eat-me signals or markers for phagocytosis, which appear on the cell surface during the apoptotic process (37, 39, 40). The engagement of eat-me signals to the corresponding receptors activates signaling pathways that ultimately generate pseudopodia, extensions of plasma membranes that surround and engulf target cells (30, 37, 40). Apoptotic cells are then incorporated, forming specialized membrane vesicles called phagosomes. Phagosomes subsequently fuse with lysosomes, giving rise to phagolysosomes (31, 34), and the components of apoptotic cells are then subjected to digestion through the actions of lysosomal enzymes. There are two partly overlapping pathways for the induction of phagocytosis, which are conserved among the nematode, fruit fly, and mammals (22, 25, 31, 34), as are those for the induction of apoptosis, and are shown in Figure 3. In the figure, the names of eat-me signals, engulfment receptors, and intracellular signal mediators of *C. elegans, Drosophila*, and mammals are shown. Phosphatidylserine (PS) is an eat-me signal common among these animal species, and transthyretin-like protein 52 (TTR52) in *C. elegans* and milk fat globule epidermal growth factor protein 8 (MFG-E8) in mammals are PS-binding proteins that bridge apoptotic cells and phagocytes. The *C. elegans* cell death abnormal protein 1 (CED-1) and its counterparts, Draper in *Drosophila* and multiple epidermal growth factor-like domains 10 (MEGF10) in mammals, and integrin INA1-PAT3 of *C. elegans* and its counterparts, αPS3–βν of *Drosophila* and α_v_–β_3_ and α_v_–β_5_ of mammals, are engulfment receptors located at the furthest upstream of the two pathways. CED-6 in *C. elegans* and its counterparts, dCed-6 in *Drosophila* and engulfment adapter protein (GULP) in mammals, and CED-2 in *C. elegans* and its counterparts, CT10 regulator of kinase (Crk) in *Drosophila*, and CrkII in mammals, are adaptor proteins that directly bind the engulfment receptors upon activation by eat-me signals. The *C. elegans* CED-5 and its counterparts, myoblast city (Mbc) of *Drosophila* and dedicator of cytokinesis 180 (Dock180) of mammals, are guanine nucleotide exchange factors that activate small G proteins. CED-12 in *C. elegans* and engulfment and cell motility (ELMO) in mammals are another adaptor proteins constituting one pathway, but their counterpart in *Drosophila*, dElmo, seems to be dispensable (41). The two pathways converge on the small G proteins CED-10 in *C. elegans*, Rac1 and Rac2 in *Drosophila*, and Rac1 in mammals, which remodel the actin cytoskeleton for the generation of pseudopodia. CED-7 in *C. elegans* and its counterparts, CG1718 in *Drosophila* and ATP-binding cassette (ABC) protein A1 in mammals, are ABC transporters whose actions in the pathways remain to be solved. Some signal mediators remain missing in these pathways and need to be identified. Other eat-me signals, bridging molecules, engulfment receptors, and signal mediators have been reported, which could be incorporated into the pathways shown here or constitute additional pathways.

## Induction of Apoptosis and Subsequent Phagocytosis of Influenza Virus-Infected Cells

**Figure 4:**
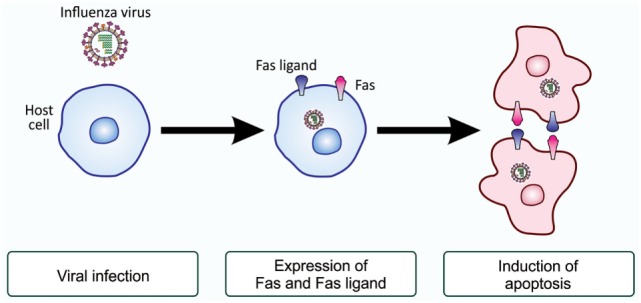
Fas and Fas ligand-induced apoptosis in influenza virus-infected cells. Upon infection with influenza virus, the production of the death receptor Fas and its ligand, the Fas ligand, is enhanced at the level of gene transcription. As a result, virus-infected cells have higher levels of Fas and the Fas ligand on their surface. When these cells associate with each other, the ligand-engaged receptor activates an intracellular signaling pathway for the induction of apoptosis. This mode of apoptosis induction is called the extrinsic pathway and does not involve mitochondria (see Figure 1).

Ranges of cell types, either primarily cultured cells or established cell lines, are susceptible to infection with influenza virus and are subsequently induced to undergo apoptosis (42–47). Previous studies demonstrated that HeLa cells and Madin–Darby canine kidney cells become apoptotic upon influenza A virus infection, characterized by the cleavage of host chromosomal DNA (48), condensation of chromatin (48), surface exposure of PS (49), and activation of initiator and effector caspases (50). Further studies demonstrated that the initiation of apoptosis in HeLa cells infected with influenza H3N2 virus may due to an elevated levels of Fas and the Fas ligand, a death receptor and its ligand (48, 49, 51). Upon infection, the activity of the transcription factor CCAAT/enhancer-binding protein β (C/EBPβ) increased, possibly through the action of double-stranded RNA-activated protein kinase (52), and this factor enhances the transcription of Fas- and Fas ligand-encoding genes (51, 53). Influenza virus-infected cells with elevated levels of Fas and the Fas ligand on the cell surface most likely interact with each other for the induction of apoptosis (54) (Figure 4). Besides the above-described study, influenza virus-induced cell death also appears to occur through the actions of apoptosis-inducing factor, another cell death-inducing ligand, in the human alveolar epithelial cell line A549, independent of caspases (55), and the upregulated expression of tumor necrosis factor-related apoptosis-inducing ligand (TRAIL) and tumor necrosis factor-α was observed in human monocyte-derived macrophages exposed to influenza H5N1 virus (56). Similarly, the induction of TRAIL was reported in natural killer cells, helper T cells, and cytotoxic T cells during infection with influenza H1N1 virus (57). Viral clearance was found to be markedly delayed in the presence of an anti-TRAIL monoclonal antibody, suggesting an important role for TRAIL in the antiviral immune response (57). A recent study demonstrated that the upregulated expression of B-cell lymphoma-2-associated X protein may be an alternative cause of the induction of apoptosis in influenza virus-infected cells (58). Nevertheless, all findings revealed that influenza virus-infected cells were induced to undergo apoptotic cell death (48, 59–61).

**Figure 5:**
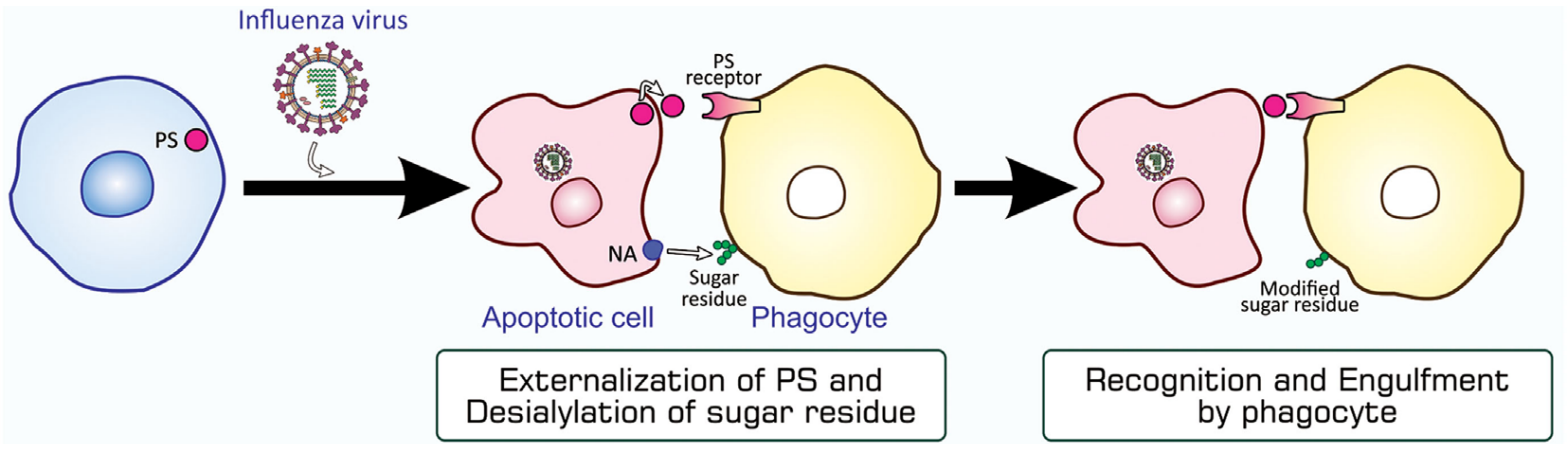
PS-mediated and sugar residue-stimulated phagocytosis of influenza virus-infected cells. Influenza virus-infected cells are induced to undergo apoptosis and express PS and viral NA on their surfaces. When phagocytes, macrophages and neutrophils, bind to these cells through interactions between PS and a PS-recognizing engulfment receptor, NA enzymatically modifies sugar residues that exist on the surface of phagocytes. The PS-bound receptor activates a signaling pathway for the induction of phagocytosis, while modified sugar residues somehow stimulate phagocytosis. NA, neuraminidase; PS, phosphatidylserine.

Although typical apoptosis mediated by caspases is evident in influenza virus-infected cells, virus replication in these cells did not appear to be impaired (48). This may be because this type of virus rapidly produces its progeny after entering host cells. In order to examine the role of apoptosis, we investigated whether influenza virus-infected cells are targeted for engulfment by phagocytes. The findings obtained from an assay for phagocytosis *in vitro* using mouse peritoneal macrophages as phagocytes showed that HeLa cells became susceptible to phagocytosis when infected with influenza A virus (62), and that this leads to the inhibition of viral propagation (63). The phagocytosis of influenza virus-infected cells appeared to be mediated by PS, the eat-me signal characterized in the most detail, and carbohydrate moieties on the surface of macrophages, which are modified by influenza virus neuraminidase expressed in virus-infected cells (62, 64) (Figure 5). Further investigations using an *in-vivo* model of infection in mice revealed the involvement of macrophages and neutrophils in the phagocytosis of cells infected with influenza A virus, and this contributed to the mitigation of influenza pathologies in mice (65). Find-me signals responsible for the recruitment of these phagocytes to the site of virus-infected cells remain to be known. The phagocytic activity of alveolar macrophages prepared from influenza virus-infected mice was stronger than that of macrophages from uninfected counterparts (65). Furthermore, the increased mortality of Toll-like receptor 4-lacking mice infected with influenza virus suggested a role for this pattern recognition receptor in antiviral mechanisms (65). The rapid mobilization of neutrophils and macrophages to target sites soon after influenza virus infection may explain the importance of pattern recognition receptors (65). Collectively, apoptosis in influenza virus-infected cells makes them susceptible to phagocytosis, and this mechanism for the direct elimination of the virus serves as an antiviral immune response.

## Induction of Apoptosis and Subsequent Phagocytosis of *Drosophila* C Virus-Infected Cells

**Figure 6:**
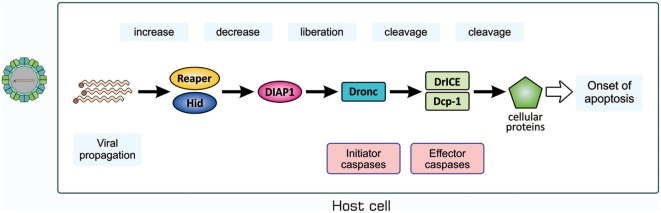
Possible mechanism for the induction of apoptosis in *Drosophila* C virus-infected cells. The process for the propagation of the virus somehow increases pro-apoptotic proteins Reaper and Hid, which cause a decrease in the level of DIAP1. This leads to the liberation and, thus, activation of the initiator caspase Dronc that partially cleaves the effector caspases DrICE and Dcp-1 for activation. Activated DrICE and Dcp-1, in turn, cleave a number of cellular proteins for apoptotic changes. The brownish dot at the end of the viral genome indicates a viral protein called virion protein, genome-linked, which plays a role in the synthesis of viral RNA. Dcp-1, death caspase-1; DIAP1, *Drosophila* inhibitor of apoptosis protein 1; DrICE, *Drosophila* interleukin-1 β-converting enzyme; Dronc, *Drosophila* Nedd2-like caspase.

We then examined whether a similar antiviral mechanism exists in insects with no adaptive immunity. We used *D. melanogaster* as a host for infection with *Drosophila* C virus (DCV), a natural pathogen of *Drosophila* (66, 67). DCV is a non-enveloped, positive-strand picorna-like RNA virus that belongs to the Dicitroviridae genus *Cripavirus* (66, 68). When S2 cells, a *Drosophila* culture cell line, were incubated with DCV, they underwent apoptosis, as was evident from chromatin condensation, DNA fragmentation, and caspase activation, accompanied by the propagation of the virus (69). We found that the amount of *Drosophila* inhibitor of apoptosis protein 1 (DIAP1), a *Drosophila* protein that inhibits caspases, decreased upon infection with DCV. All these changes in S2 cells after viral infection became undetectable when a synthetic inhibitor of caspase was present in cell cultures or the virus was pretreated with UV. The mechanisms underlying apoptosis in DCV-infected cells have not yet been elucidated; however, several studies suggested the involvement of a mechanism similar to that observed during the early developmental stages of *Drosophila* (69–71). Upon infection with Flock house virus or the DNA virus *Autographa californica* multicapsid nucleopolyhedrovirus, the expression of *reaper* and *head involution defective* (*hid*), the products of which antagonize DIAP1, was significantly increased in a manner mediated by the transcription control regions of the two genes, namely, a p53-bound sequence and sequence named the irradiation-responsive enhancer region (71). A similar mechanism appears to exist in mosquitoes when they are infected with *Culex nigripalpus* nucleopolyhedrovirus (72). We anticipate the following pathway for the induction of apoptosis in DCV-infected cells, as shown in Figure 6: the propagation of the virus enhances the transcription of *reaper* and *hid*; Reaper and/or Hid suppress DIAP1; the initiator caspase *Drosophila* Nedd2-like caspase (Dronc) is activated; Dronc cleaves and activates the effector caspases *Drosophila* interleukin-1 β-converting enzyme (DrICE) and death caspase-1 (Dcp-1); and the activated effector caspases degrade cellular proteins.

The presence of l(2)mbn cells, a *Drosophila* cell line derived from larval hemocytes, in cultures of DCV-infected S2 cells induced a decrease in the amount of the virus (69). Therefore, we examined whether DCV-infected cells are phagocytosed in a manner that is dependent on apoptosis and found that this was the case. Phagocytosis was partly inhibited in the presence of a PS-containing liposome that interferes with the action of this phospholipid as an eat-me signal. *Drosophila* phagocytes used the engulfment receptors Draper and integrin αPS3–βν for the phagocytosis of apoptotic cells (30), and the inhibited expression of either receptor by RNA interference (RNAi) caused a decrease in the level of phagocytosis. Phagocytosis was decreased further after the simultaneous RNAi of both receptors. These findings collectively suggested that DCV-infected cells are subjected to apoptosis-dependent phagocytosis by *Drosophila* phagocytes, depending on, at least partly, the eat-me signal PS and engulfment receptors Draper and integrin αPS3–βν. In order to assess this *in vivo*, we established a fatal infection of *Drosophila* adults with DCV. The findings of an assay for survivorship revealed that Draper and integrin αPS3–βν were both involved in the protection of flies from viral infection. Measurements of the viral load during infection indicated that these engulfment receptors were responsible for reducing viral propagation in adult flies. The ectopic expression of a PS-binding protein made flies more severely succumb to DCV infection and increased the viral load, confirming the PS-mediated phagocytosis of DCV-infected cells in adult flies. Hemocytes contained in the adult hemocoel appeared to be responsible for the phagocytosis of virus-infected cells. These findings indicate that the PS-mediated, Draper and integrin αPS3–βν-dependent phagocytosis of DCV-infected, apoptotic cells by hemocytes plays a role in antiviral mechanisms in *Drosophila* (see Figure 3).

In *Drosophila*, RNAi-based antiviral innate immunity has been intensely investigated (73–81). Our study now adds another mechanism for the immune response, i.e., the apoptosis-dependent, phagocytosis-based elimination of virus-infected cells. A recent study demonstrated that *Drosophila* hemocytes spread double-stranded RNA, which induces virus-specific RNAi, in the entire body of adult flies upon viral infection; therefore, cells uninfected with the virus acquired the competence for RNAi (82). The phagocytosis of virus-infected cells may make hemocytes gain a source for the production of double-stranded RNA. More importantly, the apoptosis-dependent phagocytosis of virus-infected cells serves as an antiviral mechanism in *Drosophila*, which is only equipped with innate immunity, indicating that this mechanism is an innate immune response against viral infection and common among multicellular organisms.

## Contribution of Apoptosis-Dependent Phagocytosis to Immunity Control

**Figure 7:**
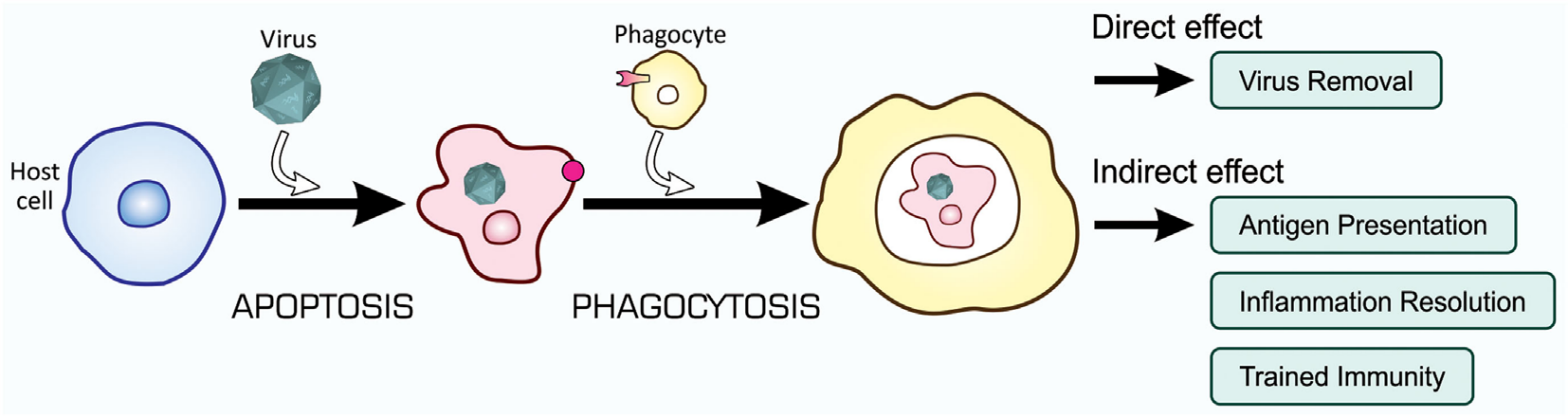
Orchestrated antiviral mechanisms initiated by the phagocytosis of virus-infected cells. Virus-infected cells are subjected to apoptosis-dependent phagocytosis, which results in the digestion of viruses together with host cells, Virus Removal. At the same time, phagocytes process incorporated viral proteins for the presentation of antigens toward CTLs for their activation, Antigen Presentation. Moreover, phagocytes change the pattern of gene expression at a transcriptional level, so that the repertoire of cytokines shifts to the mitigation of inflammation, Inflammation Resolution; the phagocytic activity of phagocytes is enhanced, Trained Immunity; and virus-specific RNAi is systemically induced, Trained Immunity. CTLs, cytotoxic T lymphocytes.

Recent studies on the mechanisms and consequences of apoptosis-dependent phagocytosis have revealed that this type of phagocytosis achieves not only the elimination of unwanted cells but also endows additional effects that contribute to the maintenance of tissue homeostasis. These effects may cooperatively control, with the direct removal of virus-infected cells, immunity to fight against viral infection, as shown in Figure 7.

### Antigen Presentation

The presentation of antigens by certain types of immune cells toward T lymphocytes is a prerequisite for the induction of adaptive immunity. Antigen presentation is mainly accomplished by dendritic cells and macrophages, antigen-presenting cells (APCs), which sample the peptides of foreign materials, such as microbial pathogens, and expose them on the cell surface together with the major histocompatibility complex (83–86). Although APCs may process the foreign peptides synthesized in these cells, the presentation of microbial antigens by APCs that are apparently not infected with the corresponding pathogens is often observed, and this is an immunological process called cross-presentation (84, 87–89). Apoptosis-dependent phagocytosis provides a mechanistic basis for this phenomenon; APCs that are not infected by the virus engulf virus-infected cells undergoing apoptosis, process viral proteins, and present viral antigens to cytotoxic T lymphocytes (CTLs) for their activation (90, 91). Activated CTLs induce apoptosis in cells that are infected with the same virus. Therefore, the phagocytosis of virus-infected cells may lead to the activation of adaptive immune responses.

### Inflammation Resolution

The phagocytic elimination of cells undergoing apoptotic cell death is an immunologically silent reaction; inflammation is not evoked. Previous research by Fadok, Henson, and others demonstrated that this mode of phagocytosis more actively resolves inflammation (92–95). They showed that macrophages engulf apoptotic neutrophils to remove cells that produce and secrete pro-inflammatory cytokines, and that neutrophil-engulfing macrophages simultaneously secrete anti-inflammatory cytokines such as transforming growth factor-β. Therefore, phagocytes alter the repertoire of cytokines after the engulfment of virus-infected cells so that possible inflammation ceases in addition to the direct elimination of virus.

### Trained Immunity

In animals equipped with innate and adaptive immunity, the first encounter with foreign substances such as microbial pathogens makes the host organism prepare for a second encounter with the same substances (96, 97). In the first encounter, the major players to combat invaders are the components of innate immunity, and they take actions not only to eliminate the invaders but also setup the activation of adaptive immunity so that adaptive responses are evoked in a rapid and robust manner in the second encounter, a mechanism called trained immunity or immunological memory. The culmination of trained immunity that involves apoptotic cell clearance is expected to be antigen presentation as described above.

Until recently, trained immunity was generally considered to not exist in innate immunity, which does not involve antibodies and lymphocytes. However, recent studies using *Drosophila* cast doubts on this assumption. Hemocytes, *Drosophila* macrophages, exhibit enhanced phagocytic activity when they encounter targets, namely, bacteria (98) and apoptotic cells (41). This is regarded as preparation for the next encounter with the same targets in order to eliminate them. The enhancement of phagocytic activity observed in both studies may have been due to a higher level of engulfment receptors. An increase of engulfment receptors in phagocytes after phagocytosis was previously reported for mammalian macrophages (96, 97), and, thus, trained immunity that leads to the activation of phagocytes is most likely conserved among multicellular organisms. Our findings also demonstrated that gene expression patterns in phagocytes changed after the engulfment of apoptotic cells, including the enhanced transcription of genes coding for engulfment receptors (41). In addition, a change in the mode of cytokine production in macrophages appears to occur at the level of transcription (99). Therefore, the mode of gene transcription appears to change in phagocytes upon the engulfment of apoptotic cells in order to more effectively control tissue homeostasis.

## Concluding Remarks

Most living organisms are always exposed to potentially fatal infections by viruses and, thus, have acquired several distinct mechanisms to prevent the invasion, proliferation, and release of viruses. The apoptosis-dependent phagocytosis of virus-infected cells is one such mechanism, through which organisms directly remove viruses from the body. Cells are induced to undergo apoptosis upon infection with a number of viruses, and this process inactivates the cellular machineries for gene expression and proliferation, which are required by invading viruses to produce their progeny in infected cells. Although apoptosis itself retards the growth of invaders, virus-infected cells appear to be equipped with a more active process, subsequent to apoptosis, for the direct elimination of viruses; virus-infected cells become susceptible to phagocytosis for degradation. Recent studies demonstrated that not only apoptotic cells, but also those undergoing other types of cell death are subjected to phagocytic elimination (100–102). Nevertheless, apoptosis remains a major biological process for the safe removal of cells unwanted by the body because it is the only mode of cell death during which the control of plasma membrane permeability is maintained. As opposed to viruses, *Leishmania major*, an intracellular parasite, appears to exploit the mechanisms described above for the establishment and dissemination of infection (103). Upon entry into animals, these types of protozoa are first captured by neutrophils that subsequently undergo apoptosis. Then, *L. major*-infected, apoptotic neutrophils are phagocytosed by macrophages, which are primary host cells for these protozoa. The pathogens survive and replicate in macrophages, and, at the same time, macrophages create an anti-inflammatory environment. As a result, *L. major* disseminates its infection. The mechanisms by which *L. major* evades killing and digestion in neutrophils and macrophages remain to be clarified.

Individual processes that constitute the apoptosis-dependent phagocytosis of virus-infected cells may be targeted by the development of novel medical treatments against virus-induced diseases. The enhancement of apoptosis in virus-infected cells may be one such treatment. Apoptosis is induced in cells that ideally need to be retained, and, thus, this treatment needs to be restricted to cells infected with viruses. In order to achieve this, the mechanisms underlying virus-induced apoptosis need to be elucidated in more detail. Another concern is the presence of proteins that inhibit apoptosis by antagonizing caspases in some types of viruses, particularly DNA viruses (20, 21, 104). The development of a method to repress apoptosis-inhibiting viral proteins may be an effective treatment. On the other hand, molecules involved in the process of the phagocytosis of virus-infected cells have been largely identified, and the stimulation of phagocytic activity is not always harmful to health. A substance that acts as an agonist for engulfment receptors is a promising candidate for an effective drug. Alternatively, the secondary effects of the phagocytosis of apoptotic cells may be targeted. The administration of apoptotic cells to patients may contribute to mitigating inflammation and stimulating phagocytes, and antiviral adaptive immunity is expected when apoptotic cells harboring viral antigens are used to treat patients. However, such efforts toward inventing novel medical treatments require great care, because the apoptosis-dependent phagocytosis of microbe-infected cells could favor the pathogens, as an example shown above.

## Author Contributions

FN mostly conducted the experiments in our study quoted in this review. FN, AS, and YN wrote the paper.

## Conflict of Interest Statement

The authors declare that the research was conducted in the absence of any commercial or financial relationships that could be construed as a potential conflict of interest.
